# Effects of Maternal Stress on Measures of Anxiety and Fearfulness in Different Strains of Laying Hens

**DOI:** 10.3389/fvets.2020.00128

**Published:** 2020-03-27

**Authors:** Mariana R. L. V. Peixoto, Niel A. Karrow, Amy Newman, Tina M. Widowski

**Affiliations:** ^1^Department of Animal Biosciences, University of Guelph, Guelph, ON, Canada; ^2^Department of Integrative Biology, University of Guelph, Guelph, ON, Canada

**Keywords:** corticosterone, layer breeder, fear, anxiety, genetics, chicken

## Abstract

Maternal stress can affect the offspring of birds, possibly due to hormone deposition in the egg. Additionally, phenotypic diversity resulting from domestication and selection for productivity has created a variety of poultry lines that may cope with stress differently. In this study, we investigated the effects of maternal stress on the behavior of different strains of laying hens and the role of corticosterone as its mediator. For this, fertilized eggs of five genetic lines—two brown (Brown 1 and 2), two white (White 1 and 2), and one pure line White Leghorn—were reared identically as four flocks of 27 birds (24F: 3M) per strain. Each strain was equally separated into two groups: Maternal Stress (“MS”), where hens were subjected to a series of daily acute psychological stressors for 8 days before egg collection, and “Control,” which received routine husbandry. Fertile eggs from both treatments were collected at three different ages forming different offspring groups that were treated as replicates; additional eggs from Control were injected either with corticosterone diluted in a vehicle solution (“CORT”) or just “Vehicle.” Eggs from each replicate were incubated and hatched, and offspring (*N* = 1,919) were brooded under identical conditions. To measure the effects of maternal stress on anxiety and fear-like behavior, offspring were subjected to a social isolation test (SI) between 5 and 10 days of age and a tonic immobility test (TI) at 9 weeks of age. Compared to Control, MS decreased the number of distress vocalizations emitted by White 2 in SI. No effects of MS were observed in TI, and no effects of CORT were observed in any tests. Overall, brown lines vocalized more in SI and remained in TI for a longer duration than white strains, suggesting genetic differences in fear behavior. Females vocalized more than males in TI and showed a trend toward significance for the same trait in SI. Overall, results suggest that the effects of maternal stress on fearfulness are not directly mediated by corticosterone. Moreover, it highlights behavioral differences across various strains of laying hens, suggesting that fear responses are highly dependent on genotype.

## Introduction

Maternal stress can impact offspring physiology, behavior, and cognition (1–3). Its effects are highly dependent on the intensity, timing of exposure, and type of stressor experienced by the mother (4–6). More specifically, impacts on offspring behavior are evident across taxa [avian (7); mammals (1, 8); reptiles (9)], and at the neurological scale, maternal stress has been linked to structural and functional changes in the limbic system and prefrontal cortex of rats (1), and to changes in gene expression in the hypothalamus of chickens (10). These brain areas are involved in the mediation of fear and anxiety, social and cognitive processes, and working memory of mammals and birds (11, 12). Maternal stress may have long-term impacts on how an animal responds to its environment. For example, in laying hens, females subjected to an unpredictable food restriction schedule had chicks that stayed longer in tonic immobility (TI), a measure of fearfulness, and were less competitive for access to food in a novel environment than the offspring of control birds (13). Similarly, the offspring of female quails stressed during egg production displayed more anxiety-like behaviors, such as an increased occurrence of distress calls during emergence and open field tests, and when isolated from conspecifics (14).

Cottrell and Seckl (15) proposed two major hypotheses to explain the association between maternal stress and postnatal effects on offspring: fetal malnutrition and overexposure to glucocorticoid hormones. More recently, studies in avian species have shown that maternal stress can also be linked to the increase in other biological components in the egg, such as androgens (16), thyroid hormones (17), antioxidants (18), and immunoglobulins (19). Nevertheless, although glucocorticoid hormones are not a synonym for “stress” (20), corticosterone remains as one of the most analyzed mediators of maternal stress in the literature due to their pleiotropic role in regulating physiological responses to the environment and in the development and maturation of vital organs [reviewed in (6, 16, 21)]. Moreover, the hypothalamus–pituitary–adrenal (HPA) axis of chickens, responsible for corticosterone production, becomes functional between the 14 and 16th day of incubation and might also be affected by maternal hormone deposition (22). The effects of corticosterone on the behavior of the offspring are, however, inconsistent and appear to depend on delivery method and species, possibly being related only to metabolic and developmental processes (23). For example, although corticosterone injections into fertile eggs and implants to female chickens were linked to an increase in the duration of TI in the offspring (24, 25), injections in yellow-legged gulls had no effects in the same test (26). Moreover, corticosterone injections decreased the offspring's ability to learn (27), compete for a wormlike object (24), and increased aggressiveness (28) in layer chickens.

As evidenced above, two experimental models are commonly used to increase corticosterone levels in the egg: a maternal model in which the adult female is exposed to stressors (either directly or through corticosterone injections or implants) and a pharmacological model that manipulates the egg. The maternal model might be considered more holistic as hormone or stress treatments integrate with other maternal elements that might also affect embryonic development (29). However, it precludes a specific control of the quantity of hormone reaching the embryo (30). Conversely, egg manipulation allows the study of exposure to an exact dose of specific hormone but relies on the use of an invasive injection procedure that can be harmful to the embryo (31, 32). Furthermore, hormonal responses are generally dose-dependent, and the actual concentrations deposited by the mother into the egg during development remain unknown (16, 33).

Similarly unknown is the relationship between maternal stress and genetics. Although no previous studies have tested multiple strains of commercial layers simultaneously, the levels of susceptibility to maternal stress may vary across different genotypes. A positive correlation between the concentration of corticosterone in layer breeders and the occurrence of an anxiety-like behavior in the offspring was observed in a white genetic hybrid but not in a brown hybrid (34). Furthermore, it has been found that adult brown and white strains of laying hens have distinct behavioral and physiological responses to stress (34–36); and comparisons between offspring of White Leghorns and their ancestor, the red jungle fowl, revealed that in response to maternal stress, only the White Leghorn chickens displayed decreased learning abilities and differences in gene expression in the hypothalamus and pituitary, suggesting that genetic selection may have increased maternal stress susceptibility (37).

The main goal of this study was to investigate whether the effects of maternal stress on offspring fear- and anxiety-like behavior differ across genetic lines of laying hens. For this, five strains of breeder hens were subjected to two stress models: one that involved subjecting the breeders to acute psychological stressors and another that involved egg injections of corticosterone. Using these treatments, we sought to decouple the role of corticosterone from the broader maternal milieu during maternal stress. We predicted that injections would affect all strains, acting as a positive control treatment regardless of genetics, and that the effects of Maternal Stress would vary according to the natural stress susceptibility of each strain.

## Materials and Methods

The birds used in this study were treated in accordance with the Canadian Council on Animal Care, and all procedures were approved by the University of Guelph Animal Care Committee (Animal Utilization Protocol #1946). All the strains presented herein were anonymized as required by the genetics companies that donated the parent stock.

### Parent Stock: Management

A total of 2,600 fertilized eggs of five strains of parent stock were provided by two commercial genetics companies (Brown 1 and White 1 from company 1; Brown 2 and White 2 from company 2; each company donated 360 female line eggs and 64 male eggs per strain) and the University of Guelph's Arkell Poultry Research Station (pure line White Leghorn). To guarantee similar experiences, eggs from all strains were collected from grandparent hens that were between 40 and 50 weeks of age. Eggs and chicks were subjected to identical incubation and husbandry conditions, as previously described (38). Chicks were wing banded at hatch, and each strain was equally distributed into 4 parent flocks that were placed in 2 rooms containing 10 pens of 27 birds (24 females and 3 males) each (see Supplementary Figure 1). Pens (3.7 m^2^) were enriched with pine shavings, one elevated perch and one lower perch, totaling a perch space of 12.8 cm/bird/pen. At 18 weeks, five nest boxes were added to each one of the pens. Chickens from different pens were visually separated from each other and did not interact at any moment. Apart from routine husbandry, all human interaction was avoided to prevent possible habituation.

### Parent Stock: Experimental Design

**Figure 1:**
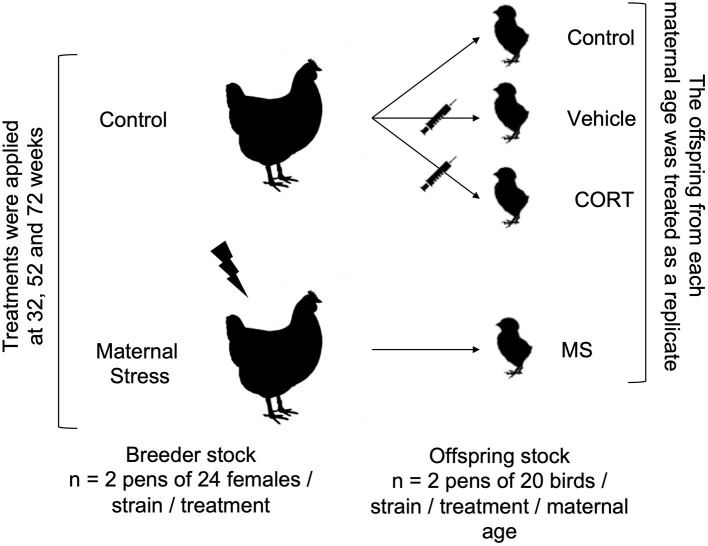
Experimental design. Treatments (Control and Maternal Stress) were applied to each strain (Commercial Brown 1 and 2, Commercial White 1 and 2, and Pure Line White Leghorn) of the breeder flocks at three different ages (32, 52, and 72 weeks). The offspring of each maternal age were statistically treated replicates.

Treatments and egg collection were performed at 32, 52, and 72 weeks of age. To form the offspring groups, equal numbers of fertile eggs (sampled over time, preference given to recent over old) from all parent flocks were incubated 1 day after the end of stressors, and the offspring flocks from each maternal age were treated as replicates (Figure 1). This experimental design allowed us to work with a larger sample size, but it also resulted in replicates confounded with incubatory settings, chick transfer and placement from the incubator to pens, and egg composition, since the nutritional value of the egg changes as a hen ages (39).

### Parent Stock: Control and Maternal Stress Treatments

Each flock of breeders was randomly assigned to either Control or Maternal Stress (“MS”) treatments with two replicate flocks per strain and treatment. Regular husbandry was strictly adhered to for the Control groups, while the females of the MS flocks were subjected to daily sessions of acute psychological stress procedures that were selected based on their ability to increase plasma corticosterone concentration in avian species (see references for each test and species below). Since the average time window for egg production from the beginning of vitellogenesis until laying is 8 days, each MS flock received a minimum of 8 consecutive days of stressors before the beginning of egg collection.

**Figure 2:**
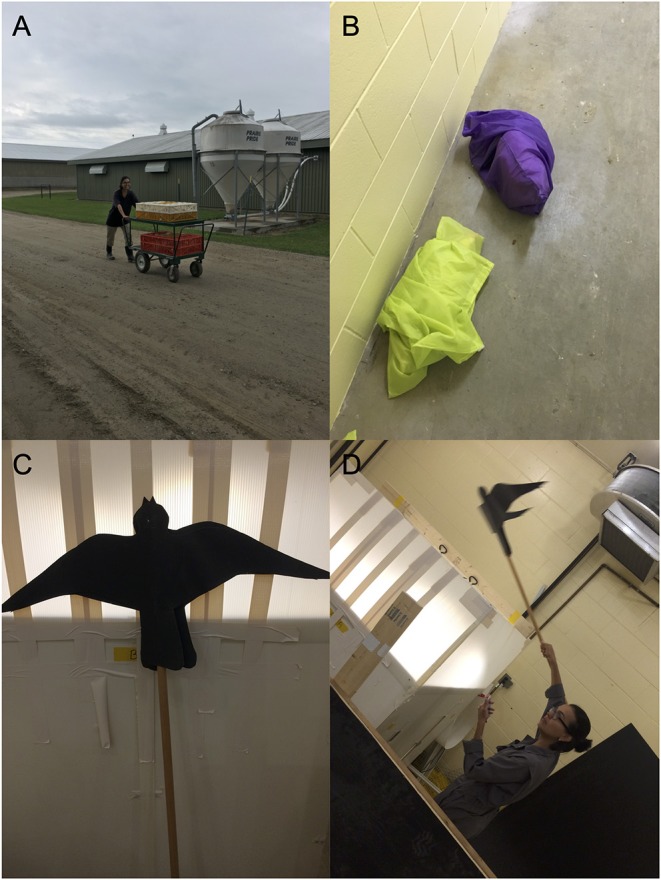
Stressors used in the MS treatment. **(A)** Breeder hens were crated and transported around the research facility for 15 min. **(B)** Physical restraint in a cloth bag. **(C)** The silhouette of a sparrow-hawk made of black cardboard (35 cm long × 50 cm wide). **(D)** Layer breeders were transferred to a test arena (100 cm long × 100 cm wide × 200 cm high); three simulations of a predator attack were performed (All photos used with permission of subjects).

Hens from the MS flocks were subjected to each of the following procedures: (1) Hens were equally distributed into two plastic crates (89 cm long × 60 cm wide × 26 cm high; 12 hens/crate), followed by 15 min of transportation [Figure 2A, laying hen: (40)]. (2) Hens were individually removed from their home pens and placed inside a cloth bag located in a nearby room for 10 min of physical restraint [Figure 2B, laying hen: (41)]. (3) Hens were crated into two groups of 12 birds, transported to an empty room 400 m away from their home pen and transferred to a test arena (100 cm long × 100 cm wide × 200 cm high) constructed of solid white panels with two doors located on opposite walls and two LED lights on the ceiling for 30 min. In the arena, hens were exposed to three simulations of a predator attack (30 s/each) using the silhouette of a sparrow-hawk made of black cardboard (35 cm long × 50 cm wide) [Figures 2C,D, great tit: (42)]. (4) Hens were crated and transported to the test arena for 15 min. An air horn was blown for 3 s at 5-min intervals [Japanese quail: 14; European starling: (43)]. (5) Hens were crated and transported to the test arena for 30 min with a different strain [laying hen: (44)]. All birds were immediately returned to their home pens after each stress session. Overall, sessions respected the following criteria: (1) Flocks were subjected to one stressor a day. (2) Stressors and egg collection were performed until the total number of eggs necessary for incubation had been collected. (3) To avoid a decrease in the physiological response to stressors due to repeated exposure, the minimum interval between the application of the same stressor was 4 days. (4) Sessions ran randomly from 9:00 to 16:00 h.

### Parent Stock: Vehicle and CORT Treatments

The CORT treatment aimed to increase the concentration of corticosterone in fertilized eggs from breeder hens. According to previous studies, the basal level of corticosterone in laying hens ranges from 0.3 to 5 ng/ml (45), reaching 30 ng/ml in response to stress (46). The concentration of corticosterone in egg yolks has been previously reported to range from 0.77 to 2.8 ng/g in Hy-Line Brown (47–49) to an average of 1.6 ng/g in Hy-Line White (47) and 2.13 ng/g in Bovan White (50) under control conditions. The mean concentration of corticosterone in eggs from unstressed birds has been previously reported as 1.17 in yolk and 1.55 ng/ml albumen (51). However, analytical validation of enzyme- and radio-immunoassay techniques showed the presence of cross-reactive substances that hamper the quantification of corticosterone in the yolk and albumen of eggs (52). Furthermore, recent work has shown that even when more precise techniques such as Celite or HPLC are conducted, they may not be sufficient [reviewed in (16)]. Therefore, since the exact concentration of corticosterone in eggs remains unknown, we followed the methodology proposed by Janczak et al. (32) and modified by Peixoto et al. (38), which was based on plasma corticosterone concentration of hens. Injections of 10 ng/ml cortisol diluted in sesame (CORT treatment) or sesame oil alone (Vehicle treatment) were used. In preparation for this procedure, a layer of approximately 0.5 mm of silicone sealant (General Electric, Boston, MA) was smeared on the basal tip of the shell (2 cm long × 1 cm wide) of a subsample of Control eggs 1 day before egg incubation; this sealant would help prevent gas exchange and contamination following perforation and injection through the shell. On the morning of each incubation day, Vehicle and CORT solutions were prepared. The average weight of egg content, which is estimated to be 90% of total egg weight (53), was 50, 50, and 59 g per hen age group; thus, a volume of 50 μl of either CORT or Vehicle solutions were injected into eggs from 32- to 52-week-old breeders, while 60 μl was injected into eggs from 72-week-old breeders. Injections were performed using a sterile 23-gage needle through a small hole that was perforated through the silicone layer using an egg piercer. Eggs from all treatments were immediately incubated.

### Offspring Stock: Management and Data Collection

Egg collection, incubation, and hatch occurred under similar conditions for all offspring groups. Chicks from each maternal age were individually wing-banded at hatch. The placement of chicks from each strain and treatment was randomized across rooms 40 pens equally distributed in four rooms (see Supplementary Table 1 and Supplementary Figures 2–4). Each pen (3.72 m^2^) was enriched with a perch (length: 155 cm) and litter floor. Each replicate in time aimed to comprise two pens with 20 birds each (10 female: 10 male) per treatment and strain; however, final densities varied due to lower hatchability of injected eggs (38). The test orders for the procedures described below were balanced across the period of the day for all flocks, strains, and treatments to minimize the effects of time and circadian rhythm on the results.

### Offspring Stock: Social Isolation (SI)

**Figure 3:**
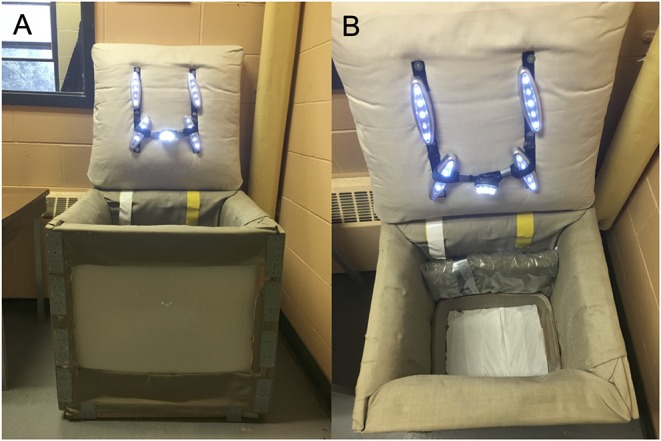
**(A)** Soundproof box (63.5 cm high × 63.5 cm deep × 63.5 cm wide) used in the social isolation test. **(B)** The box was constructed of wood panels, covered with acoustic fabric, and equipped with five LED lights and a microphone taped on the ceiling.

The social separation of young chicks from their conspecifics produces an increase in distress vocalizations and stress-induced analgesia (54), allowing for the measurement of anxiety-related behaviors. Following the methodology proposed by Sufka et al. (55), chicks between 5 and 10 days of age (*N* = 701; Table 1) were individually placed into a squared soundproof box (63.5 cm high × 63.5 cm deep × 63.5 cm wide) where their vocalizations were recorded. The box was constructed of solid panels, covered with acoustic fabric, and equipped with five LED lights and a microphone taped on the ceiling for recordings (Figures 3A,B). SI lasted 5 min and was conducted from 08:00 to 12:00 h and from 14:00 to 18:00 h in a quiet room nearby the chicks' home pen. Distress calls were recorded, saved as an MPEG-4 file using the Voice Memos application (Apple, Cupertino, USA). The total number of distress calls emitted by the chicks were counted by three observers blind to treatment using WavePad (NCH Software, Greenwood Village, USA).

**Table 1:** Number of chickens tested by treatment, strain, and maternal age (weeks) in the social isolation and the tonic immobility tests.

		**Strain**															
		**Brown 1**			**Brown 2**			**White 1**			**White 2**			**W.Leghorn**			
		**Maternal age**			**Maternal age**			**Maternal age**			**Maternal age**			**Maternal age**			**Total**
	**Treatment**	**32**	**52**	**72**	**32**	**52**	**72**	**32**	**52**	**72**	**32**	**52**	**72**	**32**	**52**	**72**	
**Social isolation**	Control	12	12	12	12	12	12	12	12	11	12	12	12	12	12	12	**179**
	Maternal stress	12	12	10	12	12	12	12	12	12	12	12	11	12	12	12	**177**
	Vehicle	12	12	11	10	12	12	12	12	12	12	12	12	11	12	12	**176**
	CORT	12	12	11	10	12	10	12	12	12	12	12	11	10	12	9	**169**
**Total**		**48**	**48**	**44**	**44**	**48**	**46**	**48**	**48**	**47**	**48**	**48**	**46**	**45**	**48**	**45**	**701**
**Tonic immobility**	Control	-	11	12	-	12	12	-	13	12	-	12	12	-	10	12	**118**
	Maternal Stress	-	12	12	-	12	12	-	11	12	-	12	12	-	11	12	**118**
	Vehicle	-	12	11	-	12	12	-	12	12	-	12	11	-	12	12	**118**
	CORT	-	12	12	-	12	12	-	12	12	-	12	12	-	9	7	**112**
**Total**		**-**	**47**	**47**	**-**	**48**	**48**	**-**	**48**	**48**	**-**	**48**	**47**	**-**	**42**	**43**	**466**

*Presented in bold are the total number of chickens tested displayed by strain and maternal age (row) and treatment (column)*.

### Offspring Stock: Tonic Immobility (TI)

A modified version of the TI methodology proposed by Jones (56) was used to measure fear in chickens at 9 weeks of age (*N* = 466; Table 1). Chickens were individually caught, moved into a quiet nearby room, and placed on their back in a V-shaped cradle, where the experimenter gently applied pressure on their sternum (57). If immobility lasted a minimum of 10 s, it was considered a successful induction. If not, up to three consecutive attempts at induction were performed. Each test lasted 10 min or until the bird stood up. Data were collected only from the offspring of hens of 52 and 72 weeks. Testing was conducted from 09:00 to 12:00 h and 13:00 to 16:00 h, and the procedure was recorded using a camcorder (Panasonic HC-V180K) that had been positioned perpendicularly to the cradle. Behavior was analyzed from videos and included duration until the bird rights itself up, the number of vocalizations emitted during the test, and the number of inductions needed to attain a successful induction. Data were analyzed by two trained observers blind to treatment.

Although the term TI implies in a state of reduced responsiveness that includes suppressed vocal behavior and intermittent periods of eye closure and muscle tremors in the extremities (58), different responses can be observed throughout the test (e.g., vocalization and head movement). As described by Rovee and Luciano (59), TI can be classified in three stages: In stages 1 and 2, distress calls can be emitted and eyes are either open or with occasional fluttering eyelids. Whereas, in stage 3, complete eye closure, no vocalizations, head bobbing, and occasional generalized body twitches are observed. Since these behaviors may vary in response to different methodologies [which can affect the validity of the test (57, 60)], data for the duration of stage 3 of TI, which will specifically be referred to as “3^rd^ stage of TI” throughout the text, were separately recorded and analyzed.

### Statistical Analyses

The Glimmix procedure of SAS 9.4 (SAS Institute, Cary, NC) was used to perform all statistical analyses. The basic statistical model in ANOVA included fixed effects of treatment (Control, MS, Vehicle and CORT), strain (Brown 1 and 2, White 1 and 2, and White Leghorn), sex, and a treatment by strain interaction. Random effects included maternal age (32, 52, and 72 weeks) and pen (10 pens) nested in room (4 rooms), with offspring bird as the experimental unit. Further pre-planned comparisons included treatment (Control vs. MS, Control vs. Vehicle, and Control vs. CORT) and white vs. brown strains. Tests for normality included Shapiro–Wilk and Anderson Darling measurements in conjunction with visual plots. When a significant strain by treatment interaction was found, analyses controlled for the multiple testing error using the percentage of false positives, which estimates the false discovery rate [FDR (61)]. Significance was declared at P < 0.05. Reliability between observers (all blind to treatment) was calculated using Kendall's Tau-b coefficient. Kendall's τ score of 1.0 is considered a perfect relationship, and a score of 0.7 is considered acceptable (62). Consequently, scores reported for SI (Kendall's τ = 0.93; *P* < 0.001) and duration of TI (Kendall's τ = 0.82; *P* < 0.001) indicate agreement among observers.

#### Social Isolation

The SI data were subjected to the basic model and log-normally transformed to meet the assumption of a normal distribution of residuals. Significance post-FDR correction was set at P < 0.005 and followed by a power analysis (alpha = 0.005). Least square (LS-) means and standard error of means (SEM) were back-transformed and are presented in the results as the average of distress vocalizations.

#### Tonic Immobility

The duration of immobility and number of vocalizations were subjected to the basic statistical model in ANOVA. To meet the assumption of a normal distribution of residuals, data for duration were subjected to a log-normal transformation, while vocalization data were transformed by the arcsine of the square root. Random effects were grouped by strain. LS-Means and standard deviation (SD) of both tests were back-transformed and are presented in the results as the average duration of TI in seconds and the average number of calls emitted during the test. The number of attempts needed for induction is presented as a percentage of birds; data were subjected to a Poisson transformation but were not normally distributed when the model included a strain by treatment interaction. Thus, a simpler statistical model containing only treatment as the fixed effect was used. Differences between LS-means were tested using a chi-square test. Due to the small number of birds induced into stage 3 of TI (*n* = 41), residuals for measurements of duration were not normally distributed when the model included a strain by treatment interaction. Therefore, a simpler statistical model containing only strain, treatment, and sex as fixed effects was used.

## Results

### Social Isolation

The number of distress calls expressed by the offspring of layer breeders was affected by strain and stress treatment (*P* < 0.001; Table 2). Chicks of the White 2 strain vocalized less when their mothers were subjected to MS compared to Control (*P* < 0.001). Similarly, MS breeders from the Brown 1, Brown 2, and White Leghorn strains produced chicks that vocalized more than White 2. Overall, brown chicks vocalized more than white (*P* < 0.001), and sex displayed a trend toward significance (*P* = 0.066), with females (125.7 ± 39.6 calls) vocalizing more than males (99.4 ± 31.3 calls).

**Table 2:** Average number of distress vocalizations (± SEM) performed by chicks between 5 and 10 days of age during the social isolation test.

**Treatment**	**Strain**					**Treatment average**
	**Brown 1**	**Brown 2**	**White 1**	**White 2**	**White leghorn**	
Control	222.3 ± 104.7^a, y^	144.2 ± 71.0^a, y^	119.8 ± 53.7^a, y^	135.3 ± 61.0^a, y^	47.7 ± 23.2^a, y^	**119.89** **±** **40.2**
Maternal stress	393.7 ± 178.0^a, y^	222.1 ± 101.1^ab, y^	60.3 ± 27.8^bc, y^	13.8 ± 6.2^c, z^	80.9 ± 38.3^ab, y^	**89.89** **±** **30.35**
Vehicle	126.0 ± 59.4^a, y^	302.7 ± 144.0^a, y^	60.3 ± 31.7^a, y^	86.3 ± 39.0^a, y^	203.7 ± 100.9^a, y^	**132.15** **±** **44.9**
CORT	118.7 ± 58.0^a, y^	220.4 ± 116.3^a, y^	63.8 ± 29.5^a, y^	44.4 ± 21.1^a, yz^	213.0 ± 110.6^a, y^	**109.56** **±** **38.0**
**Strain average**	**190.05** **±** **66.6**^**a**^	**215.01** **±** **76.90**^**a**^	**72.64** **±** **35.84**^**bc**^	**51.65** **±** **18.0**^**c**^	**113.75** **±** **58.0**^**ab**^	

*Results are presented by strain and treatment. Means in the same row with different letter superscripts (a, b, c) differ significantly between strains (P < 0.05). Means in the same column with different letter superscripts (y, z) differ significantly among treatments (P < 0.05). Presented in bold are the average number of distress vocalization per strain (row) and treatment (column)*.

### Tonic Immobility

**Figure 4:**
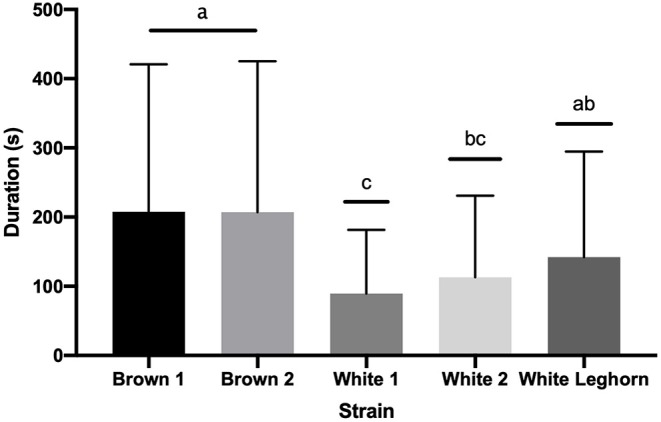
Duration (s) of tonic immobility displayed by strain (+ SD). Means with different letter superscripts^(a−c)^ differ (*P* < 0.05).

The duration of TI in 9-week-old offspring of layer breeders was not affected by an interaction of strain by treatment (*P* = 0.105), treatment (*P* = 0.924), or sex (*P* = 0.643); but brown chickens stayed longer (*P* < 0.001) in TI than white (Figure 4). The duration of the third stage of TI was not affected by treatment (*P* = 0.863), strain (*P* = 0.701), or sex (*P* = 0.089).

**Figure 5:**
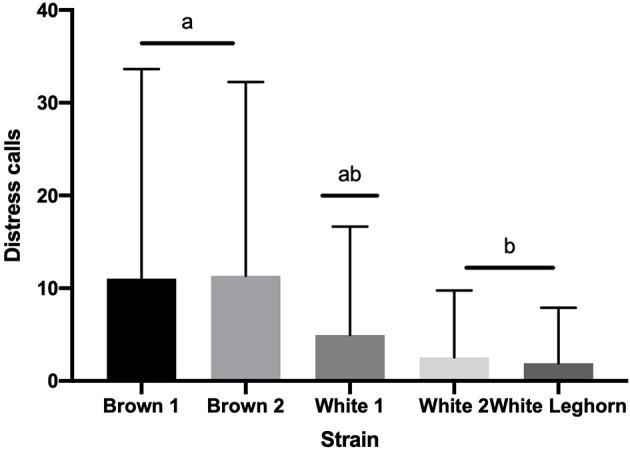
Number of distress calls (+SD) emitted during tonic immobility displayed by strain. Means with different letter superscripts^(a, b)^ differ (*P* < 0.05).

The number of vocalizations expressed by offspring in TI was also not affected by an interaction of strain by treatment (*P* = 0.580) or treatment (*P* = 0.325). However, chickens of brown strain vocalized more (*P* < 0.003) than white (Figure 5), and pullets (10.2 ± 1.2 calls) vocalized more (*P* < 0.001) than cockerels (3.05 ± 0.7 calls).

**Figure 6:**
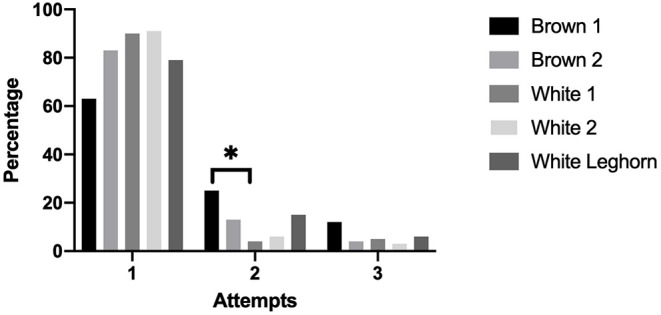
Percentage of chickens that were induced into tonic immobility after 1, 2, or 3 attempts displayed by strain (*P < 0.05).

The number of attempts needed to induce a chicken into TI was not affected by treatment (*P* = 0.892). More chickens from the Brown 1 strain needed a second attempt to reach TI compared to the White 1 strain (*P* = 0.015) (Figure 6).

## Discussion

### Limitations and Effects of the Stress Treatments

This study aimed to determine the effects of maternal stress on the behavior of different strains of laying hens. We hypothesized that the CORT treatment would show a clear response acting as a positive control treatment, while MS would highlight genetic differences among strains. In contrast to our hypothesis, the CORT treatment showed no effects on the behavior of the offspring and MS decreased the number of distress calls expressed by the offspring of White 2 mothers during SI but showed no differences in TI.

One limitation of this study is that the acute stressors used in the MS treatment were based on reports in the literature and not validated in our population of layer breeders, with the exception of the physical restraint test. The HPA axis activation of a subsample of layer breeders from all strains and treatment groups was tested at 75 weeks of age (*N* = 119). Breeders from both MS and Control treatments produced elevated concentrations of corticosterone in response to the restraint test [baseline control: 2.37 ± 0.49 ng/ml; baseline MS: 2.97 ± 0.47 ng/ml (*P* = 0.822); stress response control: 5.24 ± 0.55; stress response MS: 5.73 ± 0.55 (*P* = 0.841)] confirming that layers from the MS treatment were still physiologically responsive to restraint after repeated exposure (unpublished data). Nevertheless, we were unable to measure if this transient increase in plasma corticosterone was enough to alter the egg composition. Lastly, corticosterone has a short lifetime in chickens [~22 min (63)], and each stressor used in the study lasted a maximum of 30 min from catching until layers were returned to their home pen. Chronic stress is likely more important to signal the offspring than the short-term, acute stressors used in our experiment.

Once viewed as a successful model for testing the effects of maternal stress (6), the largely unnatural and invasive aspects of the egg injection methodology should be carefully considered. Firstly, the actual concentration of corticosterone transferred from mother to egg remains unknown (52, 64), may differ across strains (47), and can potentially overwhelm the embryo if outside of the physiological range of eggs. Indeed, as published in Peixoto et al. (38), the average hatchability for the control treatment of this study was 83%, whereas hatchability for the vehicle and control treatments were 38 and 25%, respectively. The decrease in hatchability in the vehicle treatment suggests that mechanical damage such as puncturing and disrupting eggshell membranes (which might increase the chances of pushing eggshell particles into the albumen) or the chemical composition of the vehicle affected the progeny. It is also possible that the silicone layer used to seal the hole was applied ineffectively, leaving an open hole in the shell that facilitated contamination. Lastly, the high levels of embryonic mortality in the injected groups may have created a subset of birds that were more resistant to the adverse effects of the injection, limiting the generalization of the results presented herein. Until a precise method for quantifying corticosterone in the egg and less invasive procedures are available, the efficacy of this methodology and the biological relevance of the corticosterone dosage used in the present experiment are debatable.

SI is a well-validated test that has been used as an *in vivo* preclinical screening of anxiolytic drugs (65, 66), which were shown to reverse distress vocalizations and pain-related behavior in chicks (67). Moreover, birds tested with and without the presence of a mirror confirmed the assumption that vocalizations increased due to an absence of conspecifics (68). In the present study, the offspring of the White 2 MS breeders vocalized less than the Control treatment of the same strain. To our knowledge, this is the first study that evaluated the effects of maternal stress on anxiety-like behavior through the SI test, and the results are congruent with those observed in quails tested in an open field test (14). Interestingly, the eggs from stressed quails showed higher concentrations of testosterone compared to control groups. Androgenic hormones such as testosterone are known to be important mediators of maternal effects on the behavior of the offspring (69, 70), possibly more than corticosterone [reviewed in (16, 71, 72)]. In addition, genetic differences across strains display a higher susceptibility of the White 2 strain to maternal stress compared to the other strains used in this study. Nevertheless, only minimal outcomes were observed in the progeny of the MS breeders, suggesting a higher resiliency to stressors than expected.

Tonic immobility is a state of reduced responsiveness thought to be a defense strategy used to decrease the predator's interest in the prey (73). It is induced by physical restraint, and its duration is considered a measure of fearfulness in birds (56, 57). Our lack of treatment effects in TI corroborates with Rubolini et al. (26), who injected corticosterone into fertile eggs of yellow-legged gulls. Contrary to our findings, the offspring of hens subjected to an unpredictable feeding schedule stayed longer in TI (13). This stressor, however, is not necessarily associated with increased levels of corticosterone in the egg and may be translated to the offspring via different pathways (e.g., nutrition). Also using a single egg injection of corticosterone prior to incubation, Janczak et al. (74) observed that chicks from injected eggs stayed longer in TI but only if they had been previously handled, suggesting that life experiences influence this behavioral effect of maternal stress. Interestingly, physiological studies on maternal stress and the HPA axis activation of the offspring showed that treatment effects are only observed when the offspring is also subjected to stressors (75–77). Therefore, a combination of maternal stress and life experience might be essential to trigger behavioral and physiological responses in the offspring. Our lack of treatment effects in TI might, thus, be related to a natural preservation of the phenotype of the offspring, since behavioral changes can easily become detrimental. This has important consequences for predicting and managing maternal effects in both breeder and commercial flocks, which may be regularly exposed to stressful events.

Analyses of the duration of the 3rd stage TI failed to display any effects of treatment or strain. Although the description of a bird in the 3rd stage (i.e., complete eye closure, no vocalizations, head bobbing, and occasional generalized body twitches) seems more similar to the original description of TI by Nash et al. (58), it is possible that the rigorousness of the method (which excludes birds with their eyes open and vocalizing, common behaviors during TI) may have reduced the test's ability to detect subtle behavioral differences, and therefore, it is not recommended.

### Effect of Strain

Strain effects were found in both behavior tests. Contrast statements showed that the differences were primarily associated with the phylogenetically distant brown and white strains. The brown strains vocalized more in SI and TI and showed longer durations of immobility during TI, suggesting a higher occurrence of anxious and fearful behaviors compared to the white lines. This variability might be due to the intense genetic selection for productive traits in the domestic layer or by the phylogenetic, behavioral, and physiological differences across strains (34, 36, 47, 78, 79), which might be explained by evolution and domestication. Population studies exploring genetic diversity showed that brown lines originally came from African and Mediterranean genetic clustering, whereas white lines originated from the European cluster [reviewed by (78)]. Moreover, commercial brown lines are based from the Rhode Island Red, an originally dual-purpose breed (selected for both meat and eggs) with medium genetic diversity, whereas commercial white lines are based from White Leghorn, a low genetic diversity breed (80).

Genetic selection for production traits may have also affected the behavior of chickens if the traits are correlated or genetically linked. Several quantitative trait loci (QTL) related to fear response, for example, have been found on different chromosomes in White Leghorns. More specifically, TI was associated with three different QTLs on chromosome 1 that coincide with the position of two major QTLs for growth and bodyweight (81, 82). Therefore, genetic selection for body weight may have simultaneously affected fearfulness in White Leghorn. However, data for these studies were obtained exclusively from one strain, and it would be important to measure if this is also valid for lines expressing different genetics, such as brown strains. An early study of genetic differences and behavior showed that White Leghorns chicks displayed longer duration of TI than a Production Red strain, and when the two strains were crossbred, offspring showed intermediate durations (83), supporting the hypothesis that behavioral differences between brown and white strains are genetically dependent.

Contrary to our findings on vocalization (in both SI and TI) and duration of TI, the measurement of the number of attempts to attain a successful induction in TI showed that Brown 1 needed more second attempts than White 1, therefore suggesting that for this particular trait, a brown strain was less fearful than a white strain. Overall, results on anxiety and fearfulness found in the literature are often inconsistent, and a bird's motivation to engage in certain behaviors remains unclear. For example, some studies have found that brown strains lasted longer in TI (35, 36, 84, 85) and vocalized less than white strains in an open field test (86). The interpretation of these tests is thus difficult, with a multitude of factors such as genetic selection (87), hormones (88), the environment (89), and test methodology simultaneously affecting the behavior of layers.

### Effect of Sex

In accordance with previous research (25, 41, 90), the present study did not show an interaction between treatment and sex to affect measures of anxiety and fearfulness in the offspring. Nevertheless, the current study suggests that female chickens are more anxious than males, displaying a higher frequency of distress calls during TI and a similar trend pattern in SI. These findings corroborate with Jones (86), who observed that hens were more active and vocalized more than cockerels in an open field test.

The development of sexual dimorphism in behavior is mostly related to the influence of gonadal hormones, androgens, and estrogens on the nervous system (91). Individually and combined, these hormones can organize and reorganize the neuronal circuitry involved in neuroendocrine and behavioral functions, including the serotonin system (91, 92) that is responsible for anxiety traits (93, 94). Moreover, the environment can also interact with sex to affect behavior. For example, Vallorttgara and Zanforlin (95) found that social isolation from cage companions was more stressful for female than male chickens. In the current study, birds were separated from conspecifics at both tests. Consequently, the hens might have vocalized more due to an intensified emotional response experienced during the tests.

## Conclusion

Our findings suggest that the effects of maternal stress on measures of anxiety and fearfulness were contingent on genetic strain, but only when stressors are applied directly to the mother. The lack of CORT treatment effect suggests that maternal stress may not be mediated by corticosterone. Additionally, genetic strains responded differently to both behavior tests, with brown birds displaying higher levels of fearfulness in comparison to white strains, suggesting genetic differences in fear behavior across the genetic lines of commercial layers. These findings have important implications, since behavioral variations can be decisive to determine the overall adaptability of a strain to a specific production system. Moreover, in research settings, researchers must take into consideration behavioral differences when assessing different strains of laying hens, since generalization might be misleading.

## Data Availability Statement

The datasets generated for this study are available on request to the corresponding author.

## Ethics Statement

The animal study was reviewed and approved by University of Guelph Animal Care Committee (Animal Utilization Protocol #1946).

## Author Contributions

TW conceived the work and prepared the grants. TW and MP designed the study and prepared the manuscript. NK and AN contributed to the conception of the study. MP conducted the work and analyzed the data. All authors reviewed and approved the final manuscript.

### Conflict of Interest

TW holds the Egg Farmers of Canada Chair in Poultry Welfare. The remaining authors declare that the research was conducted in the absence of any commercial or financial relationships that could be construed as a potential conflict of interest.
