# Supplementary material for: Effects of Maternal Stress on Measures of Anxiety and Fearfulness in Different Strains of Laying Hens
Source: Front Vet Sci. 2020 Mar 27;7:128. doi: 10.3389/fvets.2020.00128 (PMC7118700; doi:10.3389/fvets.2020.00128)
Supplement: Supplementary file 1 [file Data_Sheet_1.docx]

*Supplementary material.*

Table 1. Number of chicks placed in pens displayed by maternal age, strain and treatment.

| **Strain** | **Treatment** | **Maternal age (weeks)** | | | | | | | | | | |
| --- | --- | --- | --- | --- | --- | --- | --- | --- | --- | --- | --- | --- |
|  |  | **32** | | |  | **52** | | |  | **72** | | |
|  |  | **Pen 1** | **Pen 2** | **Total** |  | **Pen 1** | **Pen 2** | **Total** |  | **Pen 1** | **Pen 2** | **Total** |
| **Brown 1** | Control | 20 | 20 | 40 |  | 20 | 19 | 39 |  | 20 | 20 | 40 |
|  | Maternal Stress | 20 | 20 | 40 |  | 20 | 20 | 40 |  | 20 | 20 | 40 |
|  | Vehicle | 11 | 11 | 22 |  | 20 | 20 | 40 |  | 19 | 18 | 37 |
|  | CORT | 12 | 12 | 24 |  | 11 | 10 | 21 |  | 20 | 0 | 20 |
| **Brown 2** | Control | 20 | 20 | 40 |  | 19 | 20 | 39 |  | 20 | 20 | 40 |
|  | Maternal Stress | 20 | 20 | 40 |  | 20 | 20 | 40 |  | 20 | 20 | 40 |
|  | Vehicle | 10 | 0 | 10 |  | 20 | 20 | 40 |  | 16 | 15 | 31 |
|  | CORT | 10 | 0 | 10 |  | 15 | 0 | 15 |  | 17 | 0 | 17 |
| **White 1** | Control | 20 | 20 | 40 |  | 20 | 20 | 40 |  | 20 | 20 | 40 |
|  | Maternal Stress | 20 | 20 | 40 |  | 20 | 20 | 40 |  | 18 | 18 | 36 |
|  | Vehicle | 13 | 12 | 25 |  | 17 | 16 | 35 |  | 20 | 20 | 40 |
|  | CORT | 15 | 0 | 15 |  | 14 | 13 | 27 |  | 18 | 0 | 18 |
| **White 2** | Control | 20 | 20 | 40 |  | 20 | 20 | 40 |  | 20 | 20 | 40 |
|  | Maternal Stress | 17 | 18 | 35 |  | 20 | 20 | 40 |  | 20 | 20 | 40 |
|  | Vehicle | 18 | 0 | 18 |  | 20 | 20 | 40 |  | 20 | 20 | 40 |
|  | CORT | 11 | 12 | 23 |  | 12 | 11 | 23 |  | 17 | 16 | 33 |
| **White Leghorn** | Control | 14 | 14 | 28 |  | 20 | 20 | 40 |  | 17 | 17 | 34 |
|  | Maternal Stress | 16 | 17 | 33 |  | 20 | 20 | 40 |  | 17 | 16 | 33 |
|  | Vehicle | 11 | 0 | 11 |  | 14 | 12 | 26 |  | 12 | 11 | 23 |
|  | CORT | 10 | 0 | 10 |  | 15 | 0 | 15 |  | 10 | 0 | 10 |

| **Breeder flocks** | | | | | |
| --- | --- | --- | --- | --- | --- |
| **Room 1** | | | | | |
| White 2  Control | White 1  Control | Brown 2  M. Stress | W. Leghorn M. Stress | Brown 1  M. Stress | Empty |
|  | | | | | |
| White 1  M. Stress | Brown 1  Control | White 2  M. Stress | Empty | Brown 2  Control | W. Leghorn Control |

| **Room 2** | | | | | |
| --- | --- | --- | --- | --- | --- |
| Brown 1  Control | Empty | W. Leghorn Control | Brown 2  Control | White 2  M. Stress | White 1  M. Stress |
|  | | | | | |
| White 1  Control | White 2  Control | W. Leghorn M. Stress | Empty | Brown 2  M. Stress | Brown 1  M. Stress |

Figure 1. Room and pen distribution of the breeder flocks. Each strain was equally assigned to 2 replicates of 27 birds (24 females and 3 males) per treatment.

| **Offspring of 32 weeks breeders** | | | | | |
| --- | --- | --- | --- | --- | --- |
| **Room 1** | | | | | |
| Brown 1  CORT | White 2  Vehicle | Empty | White 1  Control | Brown 2  M. Stress | W. Leghorn  Vehicle |
|  | | | | | |
| Brown 2  CORT | White 1  M. Stress | Brown 1  Vehicle | W. Leghorn  CORT | Empty | White 2  Control |

| **Room 2** | | | | | |
| --- | --- | --- | --- | --- | --- |
| W. Leghorn Control | Brown 2  Vehicle | White 1  CORT | Empty | White 2  M. Stress | Brown 1  M. Stress |
|  | | | | | |
| W. Leghorn M. Stress | Brown 1  Control | White 2  CORT | Brown 1  Control | White 1  Vehicle | Empty |

| **Room 3** | | | | | |
| --- | --- | --- | --- | --- | --- |
| White 1  M. Stress | Brown 1  Vehicle | Brown 2  M. Stress | Empty | Empty | Empty |
|  | | | | | |
| Brown 1  Control | White 2  Vehicle | Empty | White 1  Control | Empty | Empty |

| **Room 4** | | | | | |
| --- | --- | --- | --- | --- | --- |
| W. Leghorn  M. Stress | Brown 2  Control | White 2  M. Stress | Brown 1  CORT | Empty | Empty |
|  | | | | | |
| W. Leghorn  Control | White 1  CORT | White 2  Control | Brown 1  M. Stress | Empty | Empty |

Figure 2. Room and pen distribution of the progeny of layer breeders at 32 weeks of age.

| **Offspring of 52 weeks breeders** | | | | | |
| --- | --- | --- | --- | --- | --- |
| **Room 1** | | | | | |
| Empty | W. Leghorn  Control | White 1  Vehicle | Brown 2  CORT | White 2 Vehicle | Brown 1  M. Stress |
|  | | | | | |
| Empty | White 1  CORT | Brown 2  M. Stress | W. Leghorn  Vehicle | Brown 1  Control | White 2  CORT |

| **Room 2** | | | | | |
| --- | --- | --- | --- | --- | --- |
| Empty | Brown 1  CORT | White 2 Control | W. Leghorn  M. Stress | Brown 2  Vehicle | White 1  M. Stress |
|  | | | | | |
| Empty | White 2  M. Stress | Brown 1  Vehicle | White 1  Control | W. Leghorn  CORT | Brown 2  Control |

| **Room 3** | | | | | |
| --- | --- | --- | --- | --- | --- |
| Empty | Empty | Brown 1  Control | White 1  CORT | Brown 2  M. Stress | W. Leghorn  Vehicle |
|  | | | | | |
| Empty | Brown 2  Vehicle | W. Leghorn  M. Stress | White 2  Control | White 1  M. Stress | Brown 1  CORT |

| **Room 4** | | | | | |
| --- | --- | --- | --- | --- | --- |
| Empty | Empty | Brown 1  Vehicle | Brown 2  Control | White 1  Control | White 2  M. Stress |
|  | | | | | |
| Empty | Brown 1  M. Stress | White 2  Vehicle | W. Leghorn  Control | White 2 CORT | White 1  Vehicle |

Figure 3. Room and pen distribution of the progeny of layer breeders at 52 weeks of age.

| **Offspring of 72 weeks breeders** | | | | | |
| --- | --- | --- | --- | --- | --- |
| **Room 1** | | | | | |
| Empty | White 1  Control | Brown 2  Control | W. Leghorn  CORT | White 2  M. Stress | Brown 1  Vehicle |
|  | | | | | |
| Empty | W. Leghorn  M. Stress | Brown 1  CORT | White 2  Control | White 1  M. Stress | Brown 2  Vehicle |

| **Room 2** | | | | | |
| --- | --- | --- | --- | --- | --- |
| Empty | White 2  CORT | Brown 1  Control | White 1  CORT | Brown 2  M. Stress | W. Leghorn  Vehicle |
|  | | | | | |
| Empty | Brown 2  CORT | W. Leghorn  Control | Brown 1  M. Stress | White 1  Vehicle | White 2  Vehicle |

| **Room 3** | | | | | |
| --- | --- | --- | --- | --- | --- |
| Empty | Empty | W. Leghorn  M. Stress | White 2  Control | Brown 2  Vehicle | White 1  M. Stress |
|  | | | | | |
| Empty | White 1  Control | White 2  M. Stress | Empty | Brown 2  Control | ISA Brown 1  Vehicle |

| **Room 4** | | | | | |
| --- | --- | --- | --- | --- | --- |
| Empty | Empty | Brown 1  M. Stress | W. Leghorn  Control | White 1  Vehicle | Empty |
|  | | | | | |
| Empty | Empty | Brown 2  M. Stress | White 2  Vehicle | Brown 1  Control | White 1  CORT |

Figure 4. Room and pen distribution of the progeny of layer breeders at 72 weeks of age.
